# Exploring grip strength as a predictor of depression in middle-aged and older adults

**DOI:** 10.1038/s41598-021-95566-7

**Published:** 2021-08-05

**Authors:** Adilson Marques, Duarte Henriques-Neto, Miguel Peralta, Priscila Marconcin, Élvio R. Gouveia, Gerson Ferrari, João Martins, Andreas Ihle

**Affiliations:** 1 CIPER, Faculty of Human Kinetics, University of Lisbon, Lisbon, Portugal; 2 ISAMB, University of Lisbon, Lisbon, Portugal; 3 Faculty of Human Kinetics, University of Lisbon, Lisbon, Portugal; 4 Department of Physical Education and Sport, University of Madeira, Funchal, Portugal; 5 LARSYS, Interactive Technologies Institute, Funchal, Portugal; 6 Laboratorio de Ciencias de la Actividad Física, el Deporte y la Salud, Facultad de Ciencias Médicas, Universidad de Santiago de Chile, USACH, Santiago, Chile; 7 Department of Psychology, University of Geneva, Geneva, Switzerland; 8 Center for the Interdisciplinary Study of Gerontology and Vulnerability, University of Geneva, Geneva, Switzerland; 9 Swiss National Centre of Competence in Research LIVES – Overcoming Vulnerability: Life Course Perspectives, Lausanne and Geneva, Switzerland; 10 Faculty of Human Kinetics, University of Lisbon, Estrada da Costa, 1499-002 Cruz Quebrada, Portugal

**Keywords:** Depression, Geriatrics

## Abstract

Grip strength (GS) is an indicator of health and vulnerability and inversely associated with depressive symptoms. The aim of this study was to explore GS discrimination capacity for depression; and possible GS cut-off values for depression by sex and age group. Data from 2011 and 2015 on 20,598 (10,416 women) middle-aged and older adults from 14 European countries was analysed. GS was assessed by dynamometer, and depressive symptoms using the EURO-D scale. GS cut-off values for depression were calculated and logistic regression models were used to quantify the odds of having depression in 2011 and in 2015 according to being bellow or above the cut-off value. GS had a weak discriminant capacity for depression, with the area under the curve varying between 0.54 and 0.60 (*p* < 0.001). Sensitivity varied between 0.57 and 0.74; specificity varied between 0.46 and 0.66. GS cut-off values for discriminating depression were 43.5 kg for men and 29.5 kg for women aged 50–64 years, 39.5 kg for men and 22.5 kg for women aged ≥ 65 years. Having GS above the cut-off represents significant lower odds of depression in 2011 and 4 years later, in 2015. Healthcare practitioners and epidemiologic researchers may consider the low GS cut-off values to screen for potential depression risk. However, due to its weak discriminant values these cut-offs should not be used to identify depression.

## Introduction

Muscle strength is an important indicator of health and vulnerability^1^, inversely associated with the risk of death from all causes^2^, independently of muscle mass and physical activity levels^3^. Recently, low muscle strength has been associated with poor mental health^4^, including increased depression symptoms^5–10^. However, the association between muscle strength and mental health seems to be bidirectional, once depression is also associated with functional disability and poor physical health^11,12^. Importantly, this effect is proven to be moderated by physical activity^13^.

Although some studies report a decreasing trend in the prevalence of depression in advanced ages^14–16^, estimates suggest that 10–39% of community-dwelling older adults present depression symptoms^17,18^. Also, even if the effects of age, gender, and marital status may be small^19^, older people, women, and widowed or divorced individuals still present the highest depression scores^19,20^. Due to its high prevalence, depression must be a priority of the public health system, especially regarding the older population.

Despite depression having the most negative impact on general health, compared to other chronic diseases^21^, depression symptoms are often underdiagnosed and poorly treated^22^. The main reason is that most untreated people with depression do not believe they have a condition that requires treatment^23^. This is a serious problem, as undertreated depression is associated with severely negative consequences for older adults^24^. Screening for depression must be an individualised process, to acknowledge language and cultural differences, and contemplate intellectual or cognitive impairment, co-morbid psychiatric or other illnesses, and concurrent substance abuse^25^. Finding an easier way to screen for depression symptoms could help clinical practice and epidemiologic studies to identify those at risk and provide appropriate care.

Several studies reported the significant and inversely association of grip strength (GS) and depression symptoms^5–9^. Still, the specific threshold of muscle weakness that leads to depression symptoms was never explored and, therefore, has not been identified. Identifying cut-off values of GS for discriminating the presence of depression would provide a physical measurement that can help identify those at risk for clinical practice and epidemiological studies. In this sense, the present study aimed to explore this important topic by examining the GS discrimination capacity for depression (according to a score of depression symptoms); and exploring the GS cut-off point for depression (according to a score of depression symptoms), by sex and age group, among middle-aged and older adults from 14 European countries.

## Methods

### Participants and study design

This study is based on data from the population-based Survey of Health, Aging, and Retirement in Europe (SHARE) waves 4 (2011) and 6 (2015), accessed in June 2019. SHARE is a biennial survey on ageing, collecting information about individuals aged 50 and over in several European countries and Israel. The methodology of SHARE has been previously described^26^. The recruitment sample is the responsibility of each country project participant and follows a four-stage process coordinated by the SHARE Central coordination in Munich. First, provide a Sample Design Form (SDF) containing a complete description of the chosen sampling frame and the associated sampling design. Second, the sampling proposal is evaluated and approved by the SHARE Central coordination. Third, drawing the sample according to the approved sampling design process. Finally, the country team provides a gross sample file containing the list of selected households. For this study, participants who had data on GS in 2011 and depression symptoms in 2011 and 2015 were included. The final sample comprised 20,598 participants (10,182 men, 10,416 women), from the 14 countries: Austria, Belgium, Czech Republic, Denmark, Estonia, France, Germany, Italy, Poland, Portugal, Slovenia, Spain, Sweden, and Switzerland.

Face-to-face interviews, for approximately 90 min, at the participants’ house were performed. Physical measurements were performed in the same visit. The SHARE protocol was approved by the Ethics Committee of the University of Mannheim and the Ethics Council of the Max-Planck-Society for the Advancement of Science. The study is in accordance with the Declaration of Helsinki. Written informed consent was obtained from all participants involved in the study. The datasets analysed during the current study are available in the SHARE repository: http://www.share-project.org/home0.html.

### Measures

#### Depression symptoms

Depression symptoms were assessed by the EURO-D scale. The scale was validated in an earlier European study of depression prevalence^27^. EURO-D scores range between 0 and 12, where higher scores indicate higher levels of depression. For this contribution, and based on previous studies^28^, we define clinically significant depression as a EURO-D score greater than 4.

#### Grip strength

Using a dynamometer (Smedley, S Dynamometer, TTM, Tokyo, 100 kg), GS was measured twice on each hand. Participants could be sited or standing, with their elbows at a 90° angle, the wrist in neutral position, while keeping the upper arm tight against the trunk and the inner lever of the dynamometer adjusted to the hand. Participants squeezed the dynamometer with their hand as hard as possible for 5 s. Before the assessment, participants had the opportunity to practice. Valid measurements comprised values of two measurements in one hand that differed by less than 20 kg. GS measurements with values of 0 kg or ≥ 100 kg were excluded.

#### Covariates

Covariates were the following sociodemographic variables, body composition, chronic diseases, self-perceived health, alcohol consumption, and physical activity.

Sociodemographic variables included age and education level. Age was self-reported in years and recoded into two groups: middle-aged adults (50–64 years) and older adults (≥ 65 years). Education level was assessed according to the International Standard Classification of Education-97 (ISCED-97) codes and grouped as low education level (no education or ISCED-97 codes 1 and 2), middle educational level (ISCED-97 codes 3 and 4), and high education level (ISCED-97 codes 5 and 6).

Height and weight were self-reported, and body mass index (BMI) was calculated as body mass (kg)/height^2^ (m).

For chronic diseases, participants were asked to report the presence or absence of diseases diagnosed by a medical doctor based on a list of 14 diseases. Participants could also add other medical conditions unnamed in the list^29^. This variable was coded as having none, one or more than one (multimorbidity) chronic disease. This variable was added as a covariate in the model because of the existed association between depression and chronic disease^20^. Depression is a common comorbidity among patients who experience chronic diseases^30^.

Self-perceived health was assessed with a single item question. Participants were asked, “How is your health in general?” The response options were: poor, fair, good, very good, or excellent. This single item question has been widely used in epidemiological studies^31,32^.

Participants were asked how often they drank six or more alcoholic drinks in the previous three months. Alcohol consumption was dichotomised into “at least one glass a month” and “less than one glass a month”. It is an important covariate because drinking alcohol promotes depression^33^.

Participants reported on how many days per week they practiced moderate (e.g., brisk walking, gardening, or partaking in household activities) and vigorous physical activity (e.g., hiking or participating in sports). If participants reported never participating in moderate or vigorous physical activity they were considered physical inactive.

### Statistical analysis

Data analysis was performed using IBM SPSS Statistics v.26 (SPSS Inc., an IBM Company, Chicago, Illinois, USA). Descriptive statistics [including mean, percentages and 95% confidence interval (CI)] were calculated for all variables for the total sample and each sex separately. The GS discrimination capacity for depression was analysed using 2011 data. Discrimination reflects the capability of the model to distinguish between participants with and without depression using GS values. The predictive capability of GS alone was analysed using the area under the curve (AUC), which ranges between 0.5 and 1.0 for sensible models^34^. The receiver operating characteristics (ROC) curves that created an AUC higher 0.50 for the GS were considered to have sufficient discrimination ability between participants with and without depression (95% CI were performed using the nonparametric approach)^35^. The GS cut-off values for depression were calculated using the ROC curve coordinates with Youden’s index^36^. The cut-off values represent the best trade-off between sensitivity and specificity values for GS in each sex age-group. Because GS varies significantly with age and sex, the analysis was stratified for both sex and age group (50–64 years and ≥ 65 years). Logistic regression models were used to quantify the odds of having depression in 2011) and in 2015 according to being bellow or above the GS cut-off value. All models were adjusted for age, education level, alcohol consumption, physical inactivity, chronic diseases and self-perceived health in 2011, and stratified for sex and age group (50–64 years and ≥ 65 years).

### Ethics approval and consent to participate

The SHARE protocol was approved by the Ethics Committee of the University of Mannheim and by the Ethics Council of the Max-Planck-Society for the Advancement of Science, verifying the procedures to guarantee confidentiality and data privacy.

### Consent for publication

A written informed consent was obtained from all participants involved in the study.

## Results

Participants’ characteristics are presented in Table 1. More women than men had depression (EURO-D score ≥ 4) (29.6% vs 16.5%). In addition, almost half of men (42.6%) and women (45.3%) had more than one chronic disease. Mean GS for men and women were 44.6 kg (44.4, 44.8) and 27.9 kg (27.7, 27.8), respectively.

**Table 1:** Participants characteristics, total sample and by sex.

	% or M (95% CI)		
	Total (n = 20,598)	Men (n = 10,182)	Women (n = 10,416)
Age	64.7 (65.5, 64.8)	65.1 (64.9, 65.3)	64.2 (64.1, 64.4)
Body mass index	26.8 (26.8, 26.9)	27.3 (27.2, 27.3)	26.4 (26.3, 26.5)
**Education level**			
Low	33.4 (32.8, 34.1)	33.7 (32.8, 34.6)	33.1 (32.2, 34.0)
Medium	41.4 (40.7, 42.1)	40.9 (40.0, 41.9)	41.9 (40.9, 42.8)
High	25.2 (24.6, 25.8)	25.4 (24.5, 26.2)	25.0 (24.2, 25.8)
**Chronic diseases**			
None	25.5 (24.9, 26.1)	25.8 (25.0, 26.7)	25.2 (24.4, 26.0)
One	30.6 (29.9, 31.2)	31.6 (30.7, 32.5)	29.6 (28.7, 30.0)
More than one	43.9 (43.2, 44.6)	42.6 (41.6, 43.5)	45.3 (44.3, 46.2)
**Self-perceived health**			
Poor	5.6 (5.3, 5.9)	5.9 (5.5 ,6.4)	5.3 (4.8, 5.7)
Fair	25.1 (24.5, 25.7)	24.5 (23.7, 25.4)	25.7 (24.9, 26.6)
Good	39.3 (38.7, 40.0)	39.3 (38.4, 40.3)	39.4 (38.4, 40.3)
Very good	21.0 (20.5, 21.6)	21.2 (20.4, 22.0)	20.9 (20.1, 21.6)
Excellent	8.9 (8.6, 9.3)	9.1 (8.5, 9.6)	8.8 (8.3, 9.4)
**Drinking alcohol**			
At least 1 glass a month	13.8 (13.3, 14.2)	19.3 (18.5, 20.0)	8.4 (7.9, 9.0)
Less than 1 glass a month	86.2 (85.8, 86.7)	80.7 (80.0, 81.5)	91.6 (91.0, 92.1)
**Physical inactivity**			
Other	95.3 (95.0, 95.6)	95.4 (95.0, 95.8)	95.2 (94.8, 95.6)
Never MVPA	4.7 (4.4, 5.0)	4.6 (4.2, 5.0)	4.8 (4.4, 5.2)
Grip strength (kg)	36.1 (35.97, 36.29)	44.6 (44.4, 44.8)	27.9 (27.7, 27.8)
**EURO-D**			
Score in 2011	2.20 (2.18, 2.23)	1.82 (1.78, 1.85)	2.6 (2.5, 2.6)
Score in 2015	2.19 (2.16, 2.22)	1.84 (1.80, 1.87)	2.5 (2.5, 2.6)
**Depression in 2011**			
No	76.8 (76.3, 77.4)	83.5 (82.7, 84.2)	70.4 (69.5, 71.3)
Yes	23.2 (22.6, 23.7)	16.5 (15.8, 17.3)	29.6 (27.9, 30.5)
**Depression in 2015**			
No	77.0 (76.3, 77.4)	82.9 (82.2, 83.7)	71.2 (70.4, 72.1)
Yes	23.0 (22.4, 23.6)	17.1 (16.3, 17.8)	28.8 (27.9, 29.6)

*M* mean, *CI* confidence interval, *MVPA* moderate or vigorous physical activity.

The discriminant capacity of GS for depression in 2011, including AUC, sensitivity, specificity and cut-off values, stratified by sex and age group, is presented in Table 2. AUC was between 0.54 and 0.60 (*p* < 0.001) for both sexes and age groups, indicating a weak discriminant capacity. Furthermore, sensitivity varied between 0.57 for women aged 50–64 years, and 0.74 for women older than 64. Specificity ranged between 0.46, for women aged 50–64 years, and 0.66 for men aged 50–64 years. Overall, sensitivity values were consistently higher than specificity values within each group. GS cut-off values for discriminating the presence of depression were the following: men aged 50–64, 43.5 kg; men older than 64 years, 39.5 kg; women aged 50–64, 29.5 kg; women older than 64 years, 22.5 kg.

**Table 2:** Receiver operating characteristic for handgrip strength as an indicator of not having depression.

	Men (n = 10,182)		Women (n = 10,416)	
	50–64 years old	≥ 65 years old	50–64 years old	≥ 65 years old
**Without depression**				
n	4330	4168	4065	3266
Grip strength (kg)	48.49 ± 8.68	41.29 ± 8.55	30.37 ± 6.02	25.97 ± 5.75
**With depression**				
n	841	843	1713	1372
Grip strength (kg)	46.94 ± 9.25	38.48 ± 8.75	28.66 ± 6.59	23.89 ± 6.83
*p* value	< 0.001	< 0.001	< 0.001	< 0.001
AUC (95% CI)	0.54 (0.52, 0.57)	0.58 (0.56, 0.60)	0.58 (0.56, 0.59)	0.60 (0.58, 0.62)
Grip strength cut-off for depression (kg)	43.50	39.50	29.50	22.50
Sensitivity	0.73	0.60	0.57	0.74
Specificity	0.66	0.49	0.46	0.58

*AUC* area under curve, *CI* confidence interval.

**Figure 1:**
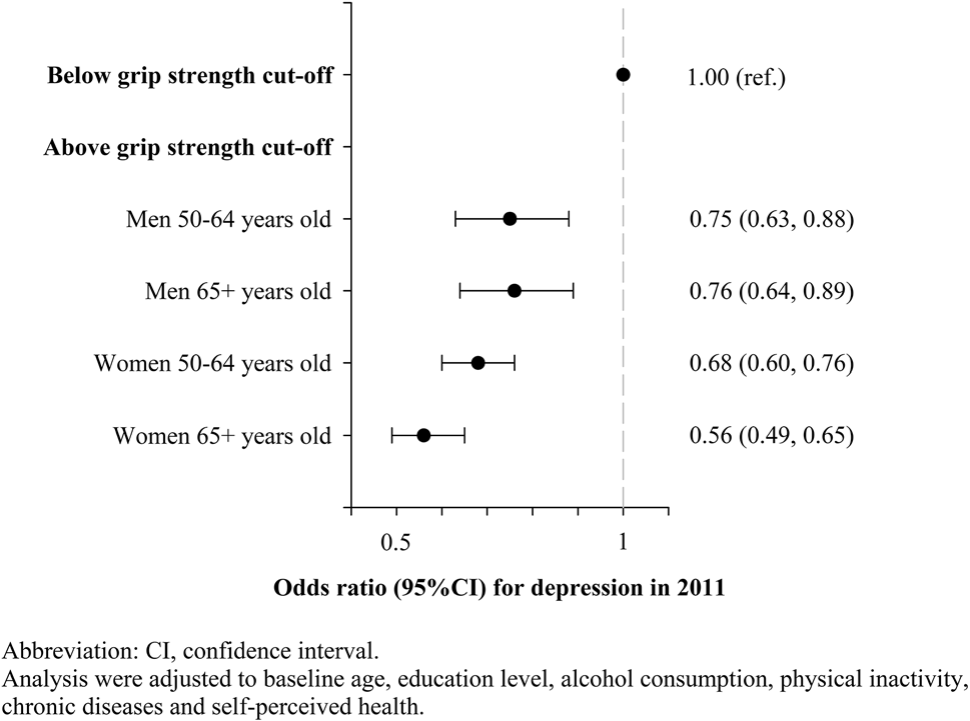
Odds ratio for having depression in 2011 according to being bellow or above the grip strength cut-off value in 2011.

**Figure 2:**
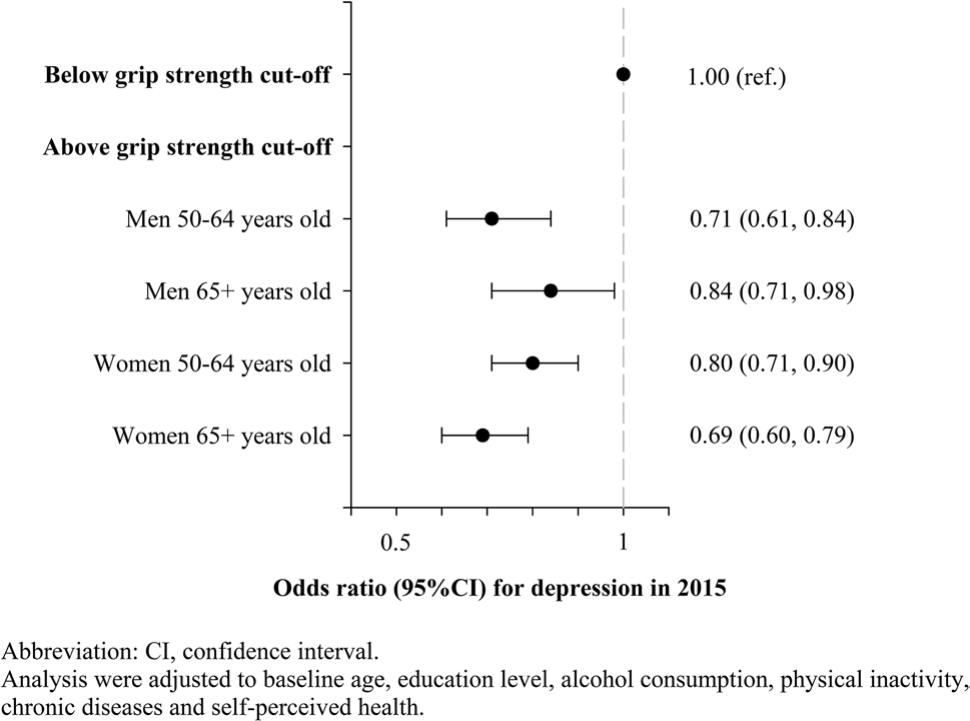
Odds ratio for having depression in 2015 according to being bellow or above the grip strength cut-off value in 2011.

Figures 1 and 2 present the odds ratio for having depression in 2011 and s in 2015 according to being bellow or above the GS cut-off value, respectively. Being above the GS cut-off value was associated with lower odds of depression in 2011 and in 2015 for men and women from both age groups. Men aged 50–64 years old and older than 64 years old above the cut-off were 25% (95% CI 0.63, 0.88) and 29% (95% CI 0.61, 0.84), and 24% (95% CI 0.64, 0.89) and 16% (95% CI 0.71, 0.98) less likely to have depression in 2011 and in 2015, respectively. Furthermore, women aged 50–64 years old and older than 64 years old above the cut-off were 32% (95% CI 0.60, 0.76) and 20% (95% CI 0.71, 0.90), and 44% (95% CI 0.49, 0.65) and 31% (95% CI 0.60, 0.79) less likely to have depression in 2011 and in 2015, respectively.

## Discussion

This study explored the GS discrimination capacity for depression symptoms by sex and age group, among middle-aged and older adults from 14 European countries. Individuals with GS values above the identified cut-off had significantly lower odds of depression in the cross-sectional and longitudinal analysis. Nonetheless, the results showed that GS had a weak discriminant capacity for depression (EURO-D score ≥ 4). The specific GS cut-off values (≤ 43.5 kg for men and ≤ 29 kg for women aged 50–64 years; and ≤ 39.5 kg for men and ≤ 22.5 kg for women older than 64 years) are not enough to screen for depression rigorously. Therefore, due to its weak discriminant values, these cut-offs should not be used to identify depression.

The prevalence of depression symptoms in 2011 was 23.2%. It was higher compared with the EURODEP study, that considered subjects aged 65 years from nine countries from Europe, the prevalence of 12.3% (95% CI 11.8–12.9)^19^. The difference could be partly explained because the EURODEP considered just high-income countries and the SHARE database include high, medium, and low-income countries. Our study also observed that women present high depressive symptoms levels compared with men (women, 29.6% vs men 16.5%), which corroborates with previous research^19,20^.

The predictive value of GS for depression, using a depression symptoms scale, in European middle-aged and older adults was identified. The AUC, sensitivity and specificity values were weak. Notwhitstanding, the analysis provided higher sensitivity values. Thus, cut-off points are prompt to have higher negative predicted values. It means that there are lower probabilities of false-negatives being detected by the GS cut-off value^37^. Therefore, when using GS for screening depression, one should be careful about the possibility of false-positive. In this line, GS may be useful as a first screening step that more specific tests should follow. In addition, other aspects of subjects should be considered, such as comorbidities, mobility limitation, physical health, and socioeconomic aspects that may influence depressive mood. Several studies already explored the potential of GS to predict health outcomes, such as the decline in activities of daily living and cognition^38^, mobility limitations^39,40^, and undernutrition screening^41^. GS seems to be a broad option for the evaluation and monitoring of health outcomes. This is justified in part because GS is strongly correlated with the overall strength^42^, and also is a simple and inexpensive assessment tool^43^.

By comparing this study’s GS cut-off values for screening depression with those of other studies focused on sarcopenia (men: < 27 kg; women: < 16 kg)^44^, and mobility limitation (men: < 25.8 kg; women: < 17.4 kg)^40^, it is possible to verify that depression cut-off values are higher. Thus, decreased GS, even before reaching a limit that indicates sarcopenia or mobility limitation, may be already indicative of worse mental health. It seems that depression affects older adults before they feel the effect of losing function and mobility. Depression among older adults cannot be justified only by physical limitations, but also by psychological and social issues related to the aging process^45^.

As reported in previous literature^19,20^, women presented a higher depression symptoms score than men. Despite that, being below the GS cut-off value was associated with higher odds of having depression in both sexes. Likewise, other studies have shown a significant association of weak GS with higher odds for depression, independently of sex^5,7,9^. On the other hand, some studies suggest this relationship to be significant only for women^46,47^. Future research should try to understand better the role of sex in the association between GS and depression, preferably using direct measures and clinical diagnosis.

Some strengths and limitations must be acknowledged. Regarding strengths, the 4-years follow up allowed to demonstrate a temporal association between low GS and higher odds of depression. Also, the analyses were stratified by sex and age groups and adjusted to several important covariates. Lastly, this study includes large representative samples from several countries, considering multiples cultures. Therefore, the external validity of the study results is a major strength. The main limitation that must be considered for future studies is that the GS assessment had some issues: there was no information about participants’ hand size and no consideration about the cultural variation of the use of grip strength in daily social routines. Also, grip strength was only measured to participants that agreed to perform this test at the data collection site. Therefore it is possible that participants undertaking this test were the ones most comfortable with it and with better grip strength, leading to potential selection bias.

## Conclusion

The following GS cut-offs are suggested for screening the risk of depression in middle-aged and older adults: ≤ 43.5 kg for men and ≤ 29.5 kg for women aged 50–64 years; and ≤ 39.5 kg for men and ≤ 22.5 kg for women older than 64 years. These cut-offs were associated with the odds of depression (EURO-D score ≥ 4 symptoms), both in 2011 and 4 years later in 2015. However, due to its weak discriminant values, these cut-offs should not be used to identify depression. Nonetheless, our findings suggest that health programs should incorporate GS in their users’ assessment because it is an outcome to assess the strength and an indicator of mental health. In addition, healthcare practitioners should be alert to the potential of depression in patients below the GS cut-off values.

## Data Availability

The datasets analysed during the current study are available in the SHARE repository: http://www.share-project.org/home0.html.
